# Diagnostic and Prognostic Potential of Circulating and Tissue BATF2 in Nasopharyngeal Carcinoma

**DOI:** 10.3389/fmolb.2021.724373

**Published:** 2021-10-28

**Authors:** Zhaolei Cui, Yingying Lin, Dan Hu, Jing Wu, Wei Peng, Yan Chen

**Affiliations:** ^1^ Laboratory of Biochemistry and Molecular Biology Research, Department of Clinical Laboratory, Fujian Medical University Cancer Hospital, Fuzhou, China; ^2^ Department of Pathology, Fujian Medical University Cancer Hospital, Fuzhou, China

**Keywords:** nasopharyngeal carcinoma, exosomes, diagnosis, prognosis, basic leucine zipper ATF-like transcription factor 2

## Abstract

**Background:** Current biomarkers for nasopharyngeal carcinoma (NPC) are less effective for early diagnosis and prognosis. The basic leucine zipper ATF-like transcription factor 2 (BATF2) gene has been shown to have a tight association with the pathogenesis of various malignancies but received scant attention in NPC research. We aimed to assess the performances of circulating and tissue BATF2 in the diagnosis and prognosis of NPC.

**Materials and Methods:** Immunohistochemistry (IHC) microarrays were performed to quantitate the BATF2 protein expression in NPC tissues. The relationships of BATF2 protein expression with clinicopathological characteristics and NPC prognosis were assessed. *BATF2* mRNA expressions in serum and serum-derived exosomes were determined using quantitative reverse transcription-polymerase chain reaction (qRT-PCR) assay.

**Results:** The IHC microarrays revealed a predominant nuclear expression of BATF2 in NPC cells. The Kaplan-Meier survival analysis showed that BATF2-positive NPC patients enjoyed longer overall survival than BATF2-negative patients. NPC patients with serum and exosomal *BATF2* mRNA expressions made up 51.47 and 48.52% of all patients, respectively. The AUCs of serum and exosomal *BATF2* mRNA expressions in discriminating NPC from healthy controls were 0.9409 and 0.8983. Patients who had received radiochemotherapy exhibited higher serum and exosomal *BATF2* mRNA expressions versus the levels at baseline as well as those detected in recurrent patients.

**Conclusion:** BATF2 is expressed cancerous tissues, serum, and serum-derived exosomes in NPC patients. Circulating and tissue BATF2 can serve as a multipurpose biomarker capable of the diagnosis, prognosis prediction, efficacy evaluation, and recurrence monitoring in NPC.

## Introduction

Nasopharyngeal carcinoma (NPC) is a head and neck malignancy originating from nasopharyngeal mucosa (Lee et al., 2019), with an incidence rate of 20–30 per 100 000 people/year (Li et al., 2011). The latest epidemiological report of NPC in China showed that the high-risk areas of NPC, where the incidence and mortality remained high in recent years, mainly included Guangdong, Guangxi, Hunan, Fujian, and Jiangxi provinces and Hong Kong (Li et al., 2011; Trejaut et al., 2011). The 10-year survival of patients is satisfactory, of 49.5–66% (Huang et al., 2015). However, the failure of early diagnosis due to concealed nasopharyngeal anatomy and atypical symptoms of early NPC remains a significant clinical concern (Ng et al., 2011). Meanwhile, nearly 50% of patients cannot achieve complete remission due to rapid tumor growth, local recurrence, or distant metastasis (Ng et al., 2011). The mechanisms for NPC carcinogenesis include a network of oncogenes and tumor-suppressor genes and the involvement of various signalings that are so complicated that not yet been fully explored (Dai et al., 2016). The known risk factors incorporate Epstein-Barr virus infection, genetic susceptibility, environmental factors, and dietary habits (Chua et al., 2016; Lin et al., 2019). At present, a biomarker for predicting NPC occurrence and recurrence and monitoring its development has not been reported (Liu et al., 2015) but will make a huge difference.

The *BATF2* gene encoding the BATF2 protein is a newly discovered tumor-suppressor gene isolated using subtractive hybridization. It is a suppressor of AP-1 regulated by interferon (SARI) or ATF-like 2 (Su et al., 2008). The *BATF2* gene maps on chromosome 11q12-11q13, and the full-length ORF shows three exons, encoding 274 amino acid residues of the BATF2 protein (Su et al., 2008). BATF2 has been reported as a tumor suppressor gene in various malignant tumors, such as lung cancer, prostate cancer, colorectal carcinoma, hepatocellular carcinoma, and nasopharyngeal carcinoma, etc (Dash et al., 2010; Huang et al., 2011; Liu et al., 2011; Ma et al., 2011; Wang et al., 2012; Chen et al., 2015; Guler et al., 2015). Aberrant BATF2 expression in prostate cancer is significantly correlated with serum PSA level, clinical stage, and distant metastasis (Chen et al., 2015). Low BATF2 expression may increase the risk of hepatocellular carcinoma (HCC) development and has a significant association with a poor prognosis (Ma et al., 2011). The same result was reported in non-small cell lung cancer (NSCLC). BATF2 deficiency promotes epithelial-mesenchymal transition (EMT), resulting in invasion and metastasis of lung adenocarcinoma cells (Wang et al., 2012). A lower *BATF2* mRNA expression was detectable in serum of patients with chronic myeloid leukemia (CML) compared to healthy controls, and *BATF2* downregulation-induced BCR-ABL inhibition was critical in the occurrence and development of CML (Huang et al., 2011). BATF2-negative colorectal cancer patients exhibited poorly differentiated cancer cells alongside deeper invasion, higher TNM stage, and shorter survival post-surgery versus BATF2-positive colorectal cancer cases (Liu et al., 2011). BATF2 downregulated the expressions of hypoxia-inducible factor (HIF-1a) and vascular endothelial growth factor *via* targeting ceruloplasmin, thus inhibiting angiogenesis and tumor growth (Liu et al., 2011). Therefore, BATF2 has been considered a prognostic indicator and a potential target for gene therapy for various cancers.

Exosomes are a type of membrane vesicles released into the extracellular matrix after fusion of multivesicular bodies with cell membranes. They are nanosized (30–150 nm) particles widely distributed in body fluids (Zeringer et al., 2015). Exosomes carry lipids, nucleic acids (such as DNA and RNA), proteins, and other components (Melo et al., 2015; He et al., 2018) and shuttle between cells, acting as important mediators of cell communication. Exosomal biomarker genes have been found a promising tool for early diagnosis and prognosis prediction in cancers (Urbanelli et al., 2015; De Rubis et al., 2019). Our previous publications have reported that serum exosomal *LDHC* overexpression can serve as a multipurpose biomarker for early diagnosis, efficacy assessment, and recurrence monitoring in breast cancer (BC) patients (Cui et al., 2020). In this study, we aimed to assess BATF2 protein expression patterns in NPC patients using IHC microarrays and the performances of circulating and tissue BATF2 in diagnosis, prognosis, and evaluation of therapeutic efficacy in NPC.

## Materials and Methods

### Clinical Data

BATF2 protein expressions in 129 NPC patients (129 tissue samples) diagnosed between January 13, 2010, and October 9, 2011, were quantified using tissue microarray (HNasN129Su01; SHANGHAI OUTDO BIOTECH). All specimens were histopathologically diagnosed. None of the patients had received radiotherapy or chemotherapy before surgical procedures. Besides, 130 serum samples and four biopsy samples of NPC tissues were collected from patients hospitalized at Fujian Cancer Hospital between January 2018 and June 2020. Fifty serum samples from healthy people were used as controls. All procedures were approved by the Ethics Committee of Fujian Cancer Hospital (Approval No. SQ2017-010-01) and written informed consent was obtained from each patient.

### IHC Microarray

The NPC microarray (HNasN129Su01) was purchased for IHC analysis of BATF2 protein expressions in NPC patients. Briefly, paraffin-embedded tissue blocks in the NPC microarray were stored overnight at 65°C, dewaxed in xylene, and rehydrated in a graded ethanol series. Endogenous peroxidase activity and nonspecific binding sites were blocked by incubating the tissue blocks in the H_2_O_2_ solution for 10 min at 37°C. Antigens were retrieved by incubation with boiling (100–120°C) citrate buffer for 3 min in a high-pressure cooker. The tissue sections were incubated in the polyclonal rabbit anti-human BATF2 antibody (Abcam, Catalog No. ab204510; 1:50) for 90 min at 37°C, washed in PBS, and incubated with the secondary antibody for 30 min at room temperature. The sections were stained with DAB for 2 min, washed in distilled water, and counterstained with hematoxylin. PBS was used instead of the primary antibody as a negative control. The samples were interpreted by two experienced pathologists. Based on the scoring criteria of our laboratory (Cui et al., 2020), the staining intensity of the NPC sections was scored based on cell color: 0 points, unstained; 1 point, light brown; 2 points, brown; 3 points, dark brown. The percentage of BATF2-positive staining was scored: 0 points, < 5%; 1 point, 5%–25%; 2 points, 25%–50%; 3 points, >50%. The final score of BATF2 expression was calculated by multiplying the percentage of BATF2-positive staining by staining intensity scores: 0 points, −; 1-2 points, +; 3-5 points, ++; 6-9 points, +++; and −/+ was regarded as low expression and ++ to +++ high expression.

### Isolation and Identification of Exosomes Derived From Serum of NPC Patients

Serum-derived exosomes were extracted with an exoRNeasy Serum/Plasma Midi Kit (QIAGEN, Catalog No.77044) according to the manufacturer’s protocol (www.qiagen.com/hb-1179). The blood samples were centrifuged at 16,000 × g for 10 min at 4°C to remove particles larger than 0.8 μm. 500 μL of serum was collected and mixed with XBP at the ratio of 1:1. The serum/XBP mix was added onto the exoEasy spin columns and centrifuged at 500 × g for 1 min. The mix was added with 3.5 ml of XWP buffer and centrifuged at 5,000 × g for 5 min 700 μL of QIAzol was added onto the membrane of the exoEasy spin column, and the samples were centrifuged at 5,000 × g for 5 min. The supernatant was pipetted out into 2-ml tubes (supplied) and added with 90 μL chloroform. The tubes were closed, vortexed for 15 s, and placed for 2–3 min at room temperature. They were centrifuged at 12,000 × g for 15 min at 4°C. The supernatant was pipetted out into new tubes and mixed with 100% ethanol at the ratio of 1:2 (v/v). The sample (>700 μL) was pipetted out and applied to the RNeasy MinElute spin column in a 2-ml collection tube (supplied) and centrifuged at ≥ 8,000 × g for 15 s at room temperature. The supernatant was discarded. 700 μL of RWT buffer was added onto the RNeasy MinElute spin column, followed by another centrifugation at ≥ 8,000 × g for 15 s at room temperature and removal of the supernatant. The step was repeated after 500 μL of RPE buffer was applied to the RNeasy MinElute spin column. The samples were centrifuged at ≥ 8,000 × g for 2 min at room temperature after 500 μL of RPE buffer was loaded onto the column. The column was then transferred to a new tube (supplied), unclosed, and centrifuged at the max speed for 5 min to dry the membrane. The RNeasy MinElute spin column was placed in a new 1.5-ml collection tube (supplied). 14 μL of RNase-free water was directly added onto the center of the spin column membrane. The column was placed for 1 min and centrifuged at the max speed for 1 min to extract RNA. The purification of the total serum RNA was assessed.

Exosomal vesicles were observed under transmission electron microscopy (TEM) (Hitachi TEM system). Exosomes were lysed in RIPA lysis buffer, and total proteins were extracted. The proteins were separated by SDS-PAGES and transferred onto a polyvinylidene difluoride membrane. After blocking, the membranes were incubated with mouse anti-human GAPDH monoclonal primary antibody (1:1,000), rabbit anti-human CD63 (1:100; ab217345, Abcam) and CD9 (1:100; ab92726, Abcam) antibodies, and rabbit anti-human BATF2 polyclonal antibody (1:300; ab204510, Abcam). Immunoblotting was performed to quantitate BATF2 protein expression in the biopsy tissues of NPC patients, as described in our previous publication (Cui et al., 2020).

### qRT-PCR Analysis

Total RNA was extracted from serum and serum-derived exosomes with a miRNeasy Kit (QIAGEN, Catalog No.217184) and an exoRNeasy Serum/Plasma Midi Kit (QIAGEN, Catalog No.77044), respectively. For extraction of total serum RNA, 200 μL of serum sample was added with 1 ml of QlAzol lysis reagent, mixed by repetitive pipetting, and placed for 5 min at room temperature. After adding 200 μL of chloroform, the mixture was vortexed for 15 s, incubated for 2–3 min at room temperature, and centrifuged at 12,000 rmp for 15 min at 4°C. The supernatant was transferred to a new EP tube, added with 100% ethanol at the ratio of 1:1.5 (v/v), and mixed by repetitive pipetting. 700 μL of the mixture was applied to the RNeasy MinElute spin column in a new 2-ml tube, centrifuged at 8,000 × g for 15 s at room temperature for nucleic acid purification. 700 μL of RWT buffer was added onto the column, followed by centrifugation (8,000 × g, room temperature) for 15 s and removal of the supernatant, which was repeated after 500 μL of RPE buffer was added. 500 μL of 80% ethanol was added onto the column. The column was centrifuged (8,000 × g, room temperature) for 2 min. The RNeasy MinElute spin column was retained and placed in a new 2-ml tube for centrifugation until the spin column membrane was dried. The column was transferred to a 1.5-mL tube, added with 14 μL of RNase-free water, unclosed, and centrifuged at the max speed for 1 min to extract RNA. Total RNA was reverse transcribed into cDNA with a Transcriptor First Strand cDNA Synthesis Kit (Roche; 04896866001). The cDNA was amplified with an SYBR Green Master Mix (ROX; REF.04913914001). The qRT-PCR was carried out using an ABI7500 real-time PCR system with two primers designed to amplify *BATF2* and *GAPDH*, respectively: *BATF2*-F: 5′-GCC​TAA​GCC​ATG​CAC​CTC​TGT-3′, *BATF2*-R: '-TCT​TCA​GCT​GCC​TTT​GTT​GCT​C -3′, *GAPDH*-F: 5′-GGA​GCG​AGA​TCC​CTC​CAA​AAT-3′, and *GAPDH*-R: 5′-GGC​TGT​TGT​CAT​ACT​TCT​CAT​GG-3’. The amplification conditions were as follows: initial denaturation at 95 °C for 10 min, then 40 cycles of denaturation at 95°Cfor 15 s, and extension at 60°C for 1 min. Serum and exosomal *BATF2* mRNA expression normalized to *GAPDH* was calculated using the 2^−ΔΔCT^ method.

### Statistical Analysis

SPSS 16.0 software was used for all statistical analyses. Normally distributed continuous variables were expressed as mean ± standard deviation (SD) unless otherwise specified. The independent-sample *t*-test was used for comparisons between groups. The *Chi*-square test or Spearman’s rank coefficient was used, as appropriate, to assess the correlations between BATF2 expression and clinicopathological characteristics. A Cox proportional hazard regression model was performed to identify risk factors for NPC survival, and the hazard ratio (HR) with its 95% confidence interval (CI) was calculated. Kaplan-Meier survival curve was applied to analyze the overall survival of NPC patients. A *P*-value of <0.05 was considered statistically significant.

## Results

### Demographic Characteristics

For the IHC analysis, the 129 NPC patients consisted of 99 males and 30 females, with a median age of 50 years (range, 20–82 years). The pathological subtypes incorporated undifferentiated non-keratinizing subtypes (112 cases), differentiated non-keratinizing squamous cell carcinoma (15 cases, including a case of non-keratinizing squamous cell carcinoma and another of keratinizing squamous cell carcinoma), and keratinizing squamous cell carcinoma (2 cases, a case of non-keratinizing squamous cell carcinoma and another of partially non-keratinizing undifferentiated subtype). Among the 129 cases, 109 did not develop distant metastasis. According to the Chinese 2017 staging system for NPC, 15 patients were diagnosed with stage I NPC, 55 with stage II NPC, 37 and 4 with stage III and IV NPC. Serum samples from 130 NPC cases were collected in our institution. The 130 NPC cases consisted of 68 newly diagnosed cases, 23 recurrent cases, and 39 cases with clinical treatment (radiotherapy or chemotherapy or targeted therapy), and their clinical characteristics are shown in Table 1.

**TABLE 1:** Clinical characteristics of the 130 NPC cases for the analysis of serum and exosomal *BATF2* mRNA expressions.

Clinicopathological features	Newly diagnosed NPC (*n* = 68)	Clinical treated NPC (*n* = 39)	Recurrent NPC (*n* = 23)
Age (years)			
>50	27	21	11
≤50	41	18	12
Gender			
Male	52	32	19
Female	16	7	4
Clinical stage			
Stage I + II	31	20	7
Stage III + IV	37	19	16
Cervical lymph nodes			
Yes	54	32	23
No	14	7	0

### Tissue BATF2 Downregulation as a Biomarker for NPC Prognosis

**FIGURE 1:**
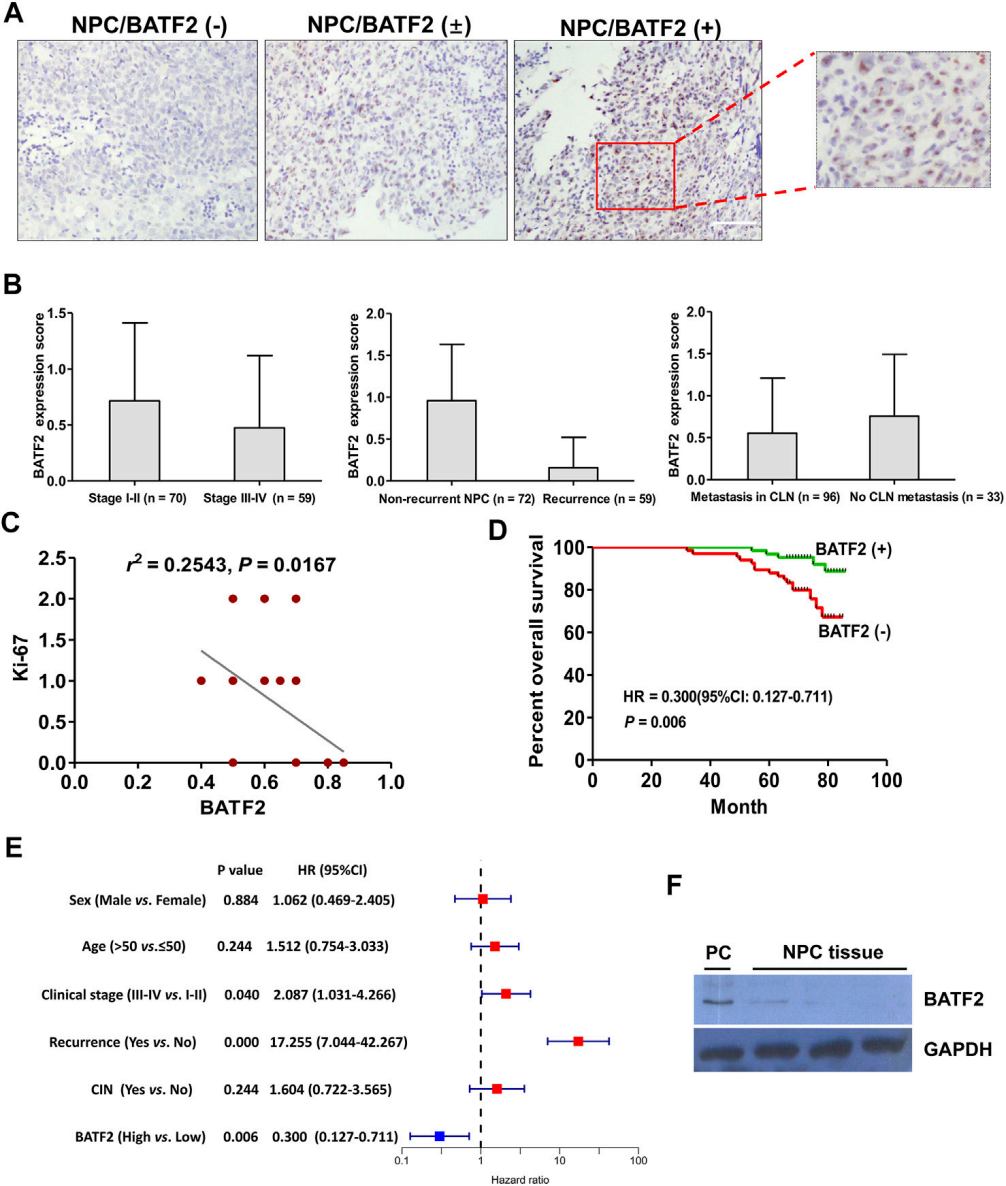
Tissue BATF2 downexpression in NPC tissues using IHC microarrays. **(A)** BATF2 is predominately expressed in the nucleus of NPC cells (× 200). **(B)** Elevated BATF2 expression scores are found in stage I-II NPC patients and those without recurrent tumor or metastasis in the cervical lymph nodes (CLNs). **(C)** Low BATF2 expression is correlated with Ki-67 upregulation in NPC tissues. **(D)** The Kaplan-Meier survival analysis shows that BATF2 expression is positively correlated with NPC prognosis. **(E)** The forest plot of risk factors for NPC prognosis (the cut-off point for HR calculation: 5-year follow-up). **(F)** Low BATF2 expression was detected in biopsy tissues using immunoblotting. The BATF2-overexpressed NPC cell line (CNE2) is utilized as a positive control (PC).

The IHC microarray showed that 63/129 (48.83%) cases were BATF2-positive, with BATF2 predominately expressed in the nucleus of NPC cells (Figure 1A) and elevated BATF2 expression scores in stage I-II NPC patients and those without recurrent tumor or metastasis in the cervical lymph nodes (Figure 1B). Correlations between BATF2 expressions and clinical characteristics in NPC patients are shown in Table 2. BATF2 expression had significant associations with clinical stage (*p* = 0.040), recurrence (*p* = 0.000), and Ki-67 expression (a prognostic biomarker) in NPC (Figure 1C; Table 2). The Kaplan-Meier survival analysis showed that BATF2-positive patients tended to have a better prognosis, whose overall survival (OS) was significantly longer versus BATF2-negative patients (Figure 1D). These findings suggested that BATF2 had the potential to predict NPC prognosis. The Cox hazards model demonstrated that advanced clinical staging, recurrence, and low BATF2 expression were risk factors for the prognosis of NPC patients (Figure 1E). A low or negative BATF2 expression was also observed in biopsy tissues using immunoblotting (Figure 1F).

**TABLE 2:** Correlations between BATF2 protein expressions and clinical characteristics in NPC patients.

Clinicopathological features	Total case size	BATF2 expression (+)	BATF2 expression (-)	χ2 value	*P* Value
Age (years)				0.533	0.244
>50	60	26	34		
≤50	69	37	32		
Gender				0.021	0.884
Male	99	48	51		
Female	30	15	15		
Recurrence				46.735	0.000
Yes	57	9	48		
No	72	55	17		
Clinical stage				4.225	0.040
Stage I + II	70	40	30		
Stage III + IV	59	23	36		
Tumor size (cm)				0.477	0.490
≥2	12	7	5		
<2	117	56	61		
Cervical lymph nodes				1.355	0.244
Yes	96	44	52		
No	33	19	14		

### Serum and Exosomal *BATF2* mRNA as a Diagnostic Biomarker for NPC

**FIGURE 2:**
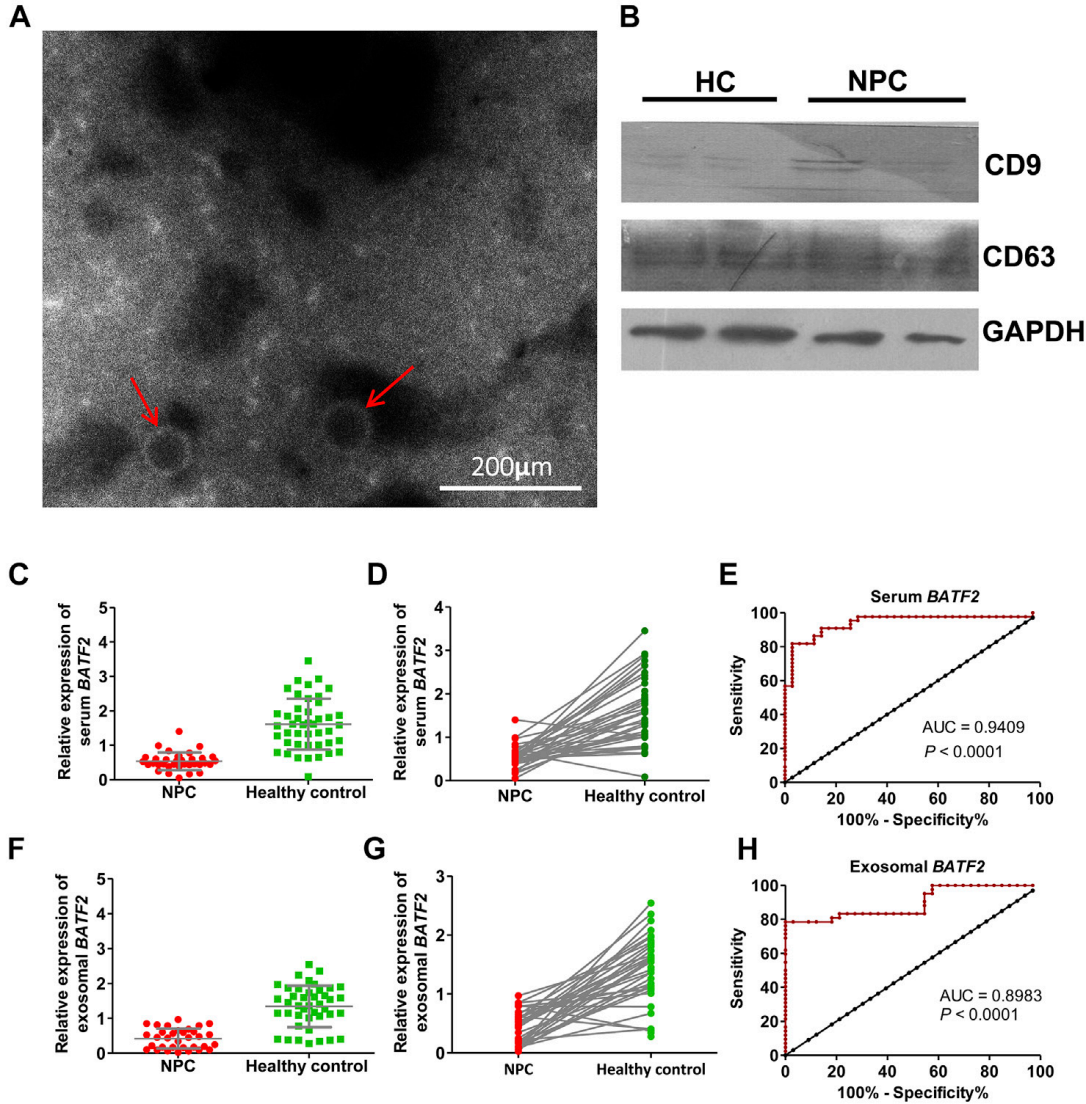
Identification of serum exosomes and efficacy of serum and exosomal *BATF2* expression in NPC diagnosis. **(A)** Morphology of serum-derived exosomes under transmission electron microscopy. The red arrows indicate isolated vesicles. **(B)** The immunoblotting assay shows that the exosome-specific markers, CD9 and CD63, are detectable in isolated vesicles. HC: healthy control. **(C,D)** Serum *BATF2* mRNA expression significantly decreases in NPC versus healthy controls. **(E)** The ROC curve to assess the efficacy of serum *BATF2* mRNA in NPC diagnosis. **(F,G)** Suppressed exosomal *BATF2* mRNA expression in NPC patients. **(H)** The ROC curve to assess the efficacy of exosomal *BATF2* mRNA in NPC diagnosis.

The morphology of serum exosomes was observed, and their size was measured under transmission electron microscopy (TEM). As shown in Figure 2A, serum exosomes were small vesicles of 30 and 150 nm in diameter with cell membrane structures. Expressions of CD9 and CD63, exosome-specific markers, were detectable in isolated vesicles (Figure 2B). We found that the percentages of patients with detectable serum and exosomal *BATF2* mRNA expression were 51.47% (35/68) and 48.52% (33/68), significantly lower compared to 88.00% (44/50) and 84.00% (42/50) in healthy controls. Both serum and exosomal *BATF2* mRNA expressions were one-third lower than those detected in healthy controls (Figures 2C,D,F,G). Subsequently, we assessed the performances of serum and exosomal *BATF2* in NPC diagnosis using ROC curves analysis and found that the sensitivity, specificity, and AUCs of serum *BATF2* in discriminating NPC patients from healthy controls were 89%, 86%, and 0.9409, respectively (Figure 2E). The sensitivity, specificity, and AUCs for exosomal *BATF2* were 81%, 82%, and 0.8983, respectively (Figure 2H). Therefore, serum and exosomal *BATF2* mRNA expressions have excellent performances in NPC diagnosis.

### Relationship of Serum and Exosomal *BATF2* mRNA Downregulation With Clinicopathological Risk Factors in NPC

**FIGURE 3:**
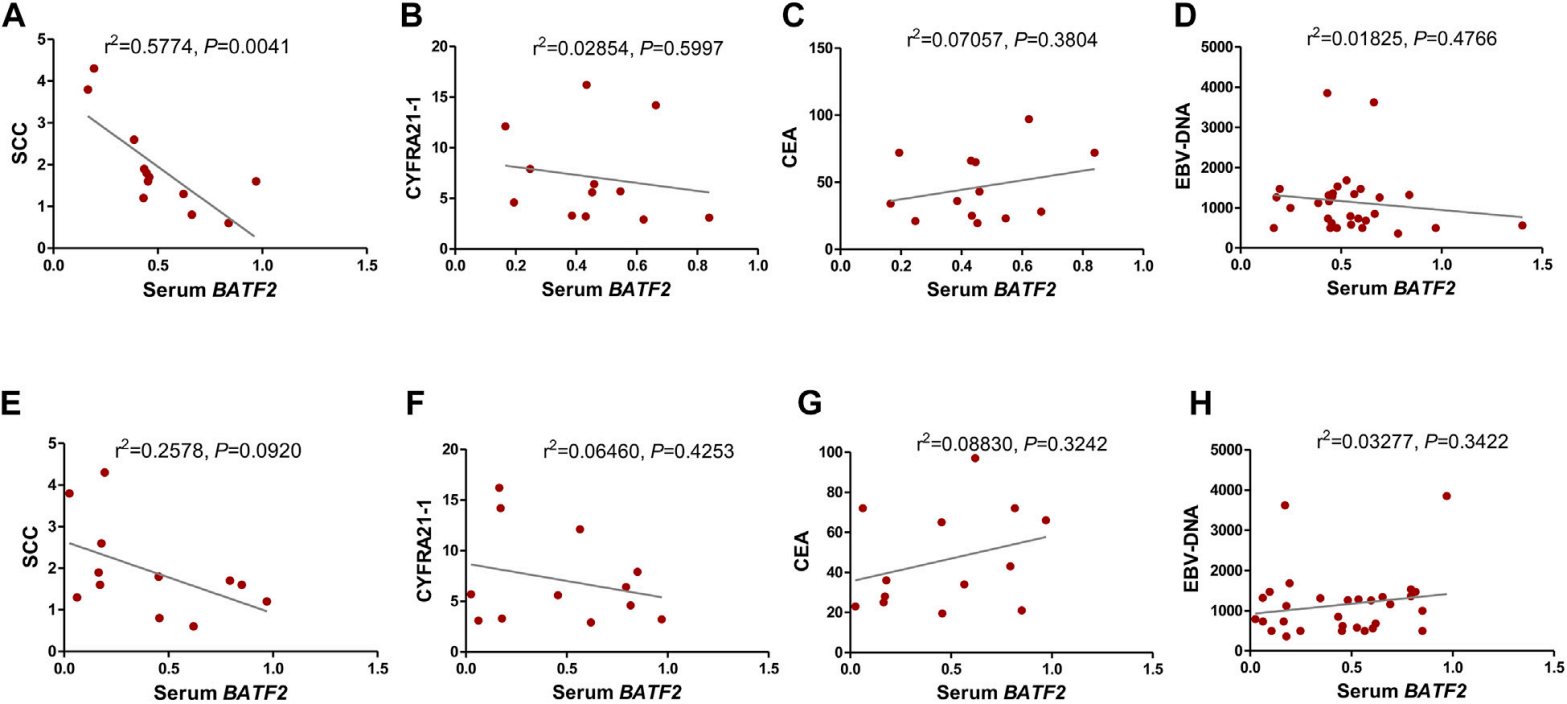
Relationship of serum and exosomal *BATF2* mRNA downregulation with clinicopathological risk factors in NPC. The correlations between serum *BATF2* expression and **(A)** SCC-Ag, **(B)** CYFRA21-1, **(C)** CEA, and **(D)** EBV-DNA levels are analyzed. The downregulation of exosomal *BATF2* expression is not associated with **(E)** SCC-Ag, **(F)** CYFRA21-1, **(G)** CEA, or **(H)** EBV-DNA levels in NPC patients.

We found that serum *BATF2* suppression had a strong association with squamous cell carcinoma antigen (SCC-Ag) levels (Figure 3A), without significant correlations with CEA, CYFRA21-1, or EBV-DNA levels (Figures 3B–D, all *p* > 0.05). However, exosomal *BATF2* downregulation was associated with none of the clinical indicators above (Figures 3E–H, all *p* > 0.05).

### Serum and Exosomal *BATF2* as an Indicator for Monitoring Efficacy and Recurrence

**FIGURE 4:**
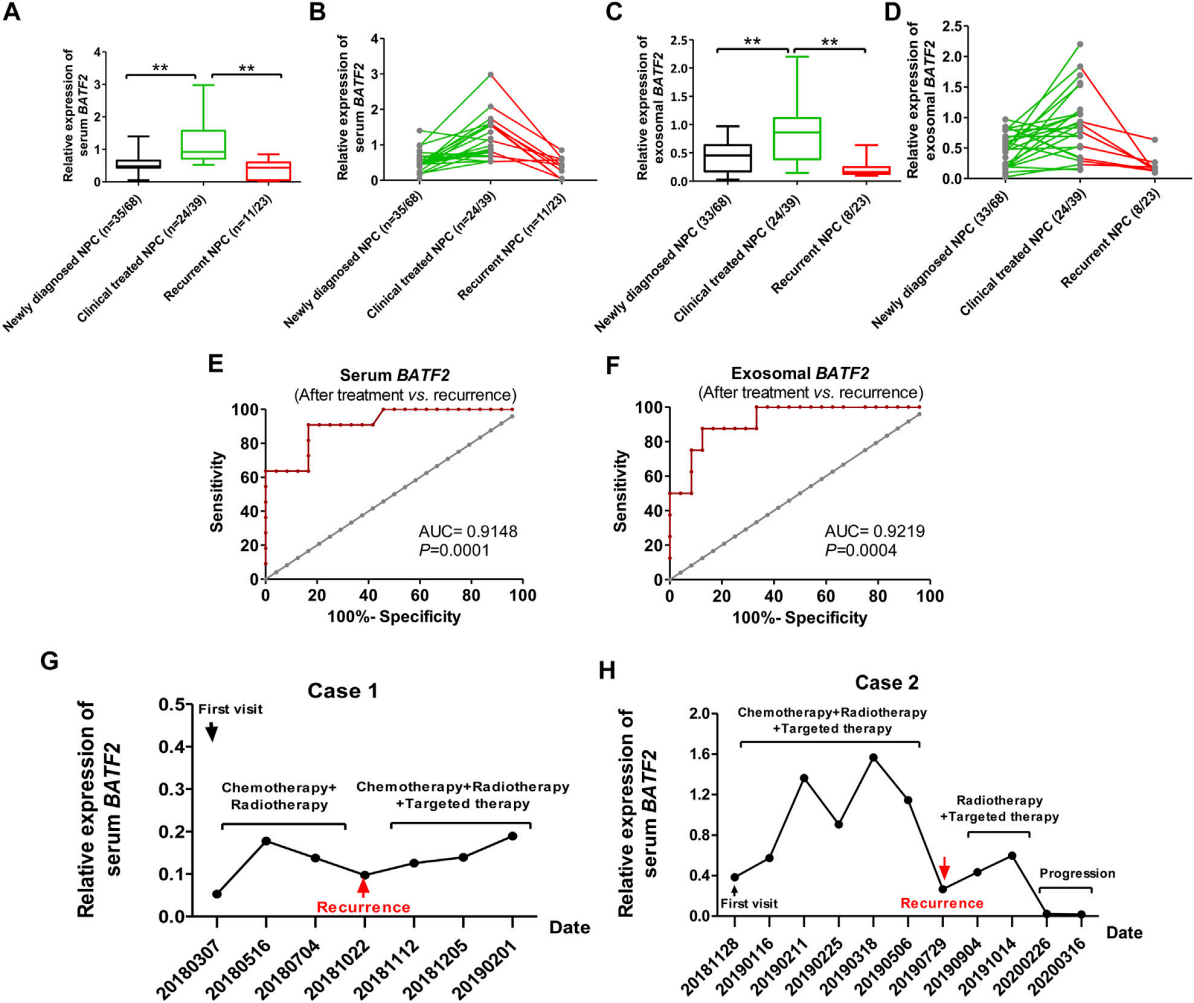
Serum and exosomal *BATF2* downregulation as an indicator for monitoring efficacy and recurrence in NPC. of Serum **(A,B)** and exosomal **(C,D)** *BATF2* mRNA expressions in NPC patients at baseline, after treatment (radiotherapy or chemotherapy or targeted therapy), at the diagnosis of recurrent NPC. ROC curves of serum **(E)** and exosomal **(F)** *BATF2* mRNA expressions in predicting the relaps eof NPC. **(G,H)** Serum *BATF2* mRNA expression level in two NPC patients participating in the follow-up during radiotherapy or chemotherapy or targeted therapy. **p < 0.01.

We assessed serum and exosomal *BATF2* expressions in NPC patients at baseline (newly diagnosed), after receiving clinical treatments (radiotherapy and/or chemotherapy and/or targeted therapy), and suffering recurrence. The results showed that serum and exosomal *BATF2* mRNA expression significantly increased after clinical treatments (*p* < 0.01), but decreased in recurrent patients (*p* < 0.01) (Figures 4A–D). Serum and exosomal *BATF2* had high AUCs in predicting the recurrence of NPC cases (Figures 4E,F). Moreover, two cases were followed up to 16 months during radiotherapy or chemotherapy or targeted therapy: their serum and exosomal *BATF2* mRNA expressions were upregulated following clinical treatments but decreased at the diagnosis of recurrent NPC (Figures 4G,H). These findings suggest serum and exosomal *BATF2* mRNA expressions as a sensitive indicator for monitoring efficacy and recurrence in NPC patients.

## Discussion

Low BATF2 expression has been shown to have a significant association with the occurrence and prognosis of various metastatic and invasive malignancies (Dash et al., 2010; Huang et al., 2011; Liu et al., 2011; Ma et al., 2011; Wang et al., 2012; Chen et al., 2015; Guler et al., 2015). For far too long, BATF2 expression patterns in NPC have not been reported. The present study reported a percentage of 48.83% of BATF2-positive patients who exhibited longer survival than BATF2-negative patients. This suggests the prognostic value of BATF2 in NPC. We found that serum *BATF2* mRNA expression was positively associated with SCC-Ag levels, which also indicates its diagnostic potential in NPC. However, aberrant BATF2 expression patterns vary among malignancies. Our previous study reported a moderate level of BATF2 expression in the K562 leukemia cell line and BATF2 downregulation-induced apoptosis and proliferation inhibition in K562 cells (Fu et al., 2018). But high BATF2 expression has been proven an important prognostic indicator for HCC (Ma et al., 2011) and NSCLC (Wang et al., 2012).

**FIGURE 5:**
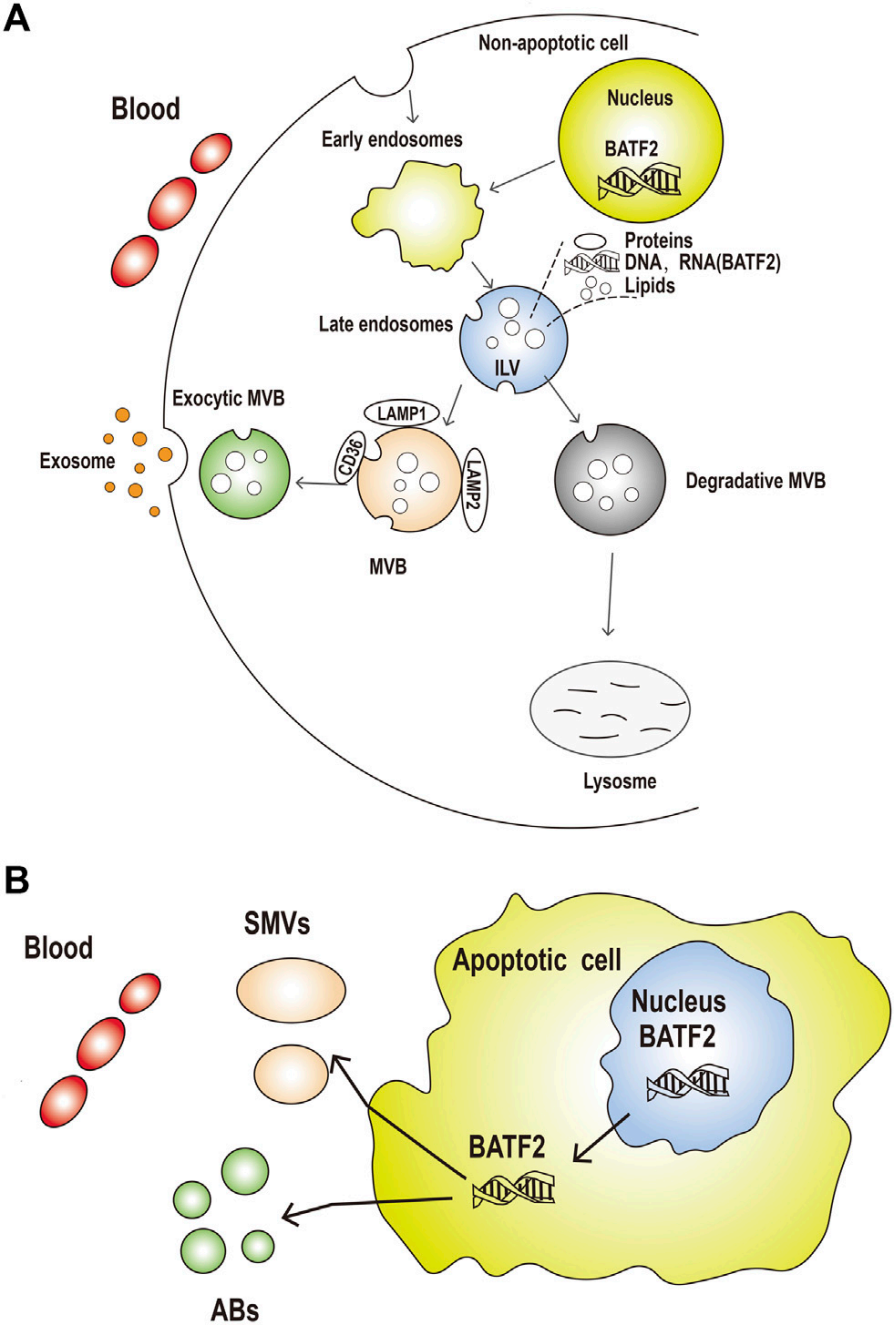
The potential mechanisms for exosomal *BATF2* secretion into the circulation. **(A)** The process of released exosomes and shed microvesicles (SMVs) (containing *BATF2* molecules) into the circulation. Early endosomal contents (proteins and nucleic acids) are either recycled to the plasma membrane (PM) or sequestered in intraluminal vesicles (ILVs), generated by the budding of the limiting membrane into the lumen of endosomes, within the larger large multivesicular bodies (MVBs). **(B)** Apoptotic or non-apoptotic dying cells result in the generation of apoptotic bodies (Abs). Microvesicles are shed from the blebbing PM or released from apoptotic cells. These vesicles are remnants of the degrading apoptotic cell with nuclear and cytoplasmic content (including *BATF2* molecules).

Exosomes are a type of nanosized extracellular vesicles exocytosed by all cells (Zeringer et al., 2015) to carry different molecules (proteins, DNAs, RNAs, or lipids) to the cells of interest (Melo et al., 2015; He et al., 2018). Exosomes are detectable in the tumor microenvironments, which have been proven to promote tumorigenesis and metastasis via promoting angiogenesis and tumor-specific immunity (Kalluri, 2016). Circulating exosomes as a cancer liquid biopsy are accessible and non-invasive for early diagnosis and allow repeated measurements for monitoring efficacy in post-treatment patients (Urbanelli et al., 2015; De Rubis et al., 2019). The present study showed that serum and exosomal expressions of cell-free *BATF2* mRNA were significantly higher in healthy controls than NPC cases, indicating the involvement of exosomal transport of *BATF2* mRNA. Current views on the potential mechanisms for exosomal RNA transfer into the circulation, as shown in Figure 5, are that exosomal *BATF2* mRNA may come both from non-apoptotic and apoptotic cells. Exosomal *BATF2* molecules may release into the extracellular median *via* exocytic fusion of multivesicular bodies (MVBs) with the plasma membrane (Mathivanan et al., 2010), followed by budding of vesicles directly from the plasma membrane and microvesicle (containing BATF2) shedding (Figure 5A). The shed microvesicles (SMVs) from apoptotic cells are called apoptotic blebs (ABs) (Figure 5B). In our study, low BATF2 expression in NPC tissues indicates its tumor suppressor roles in NPC, which is further evidenced by prolonged overall survival of BATF2-positive patients. *BATF2* mRNA expression was also detectable in serum and serum-derived exosomes of the NPC cases. Thus, we speculate that the tumor-suppressor BATF2 molecules are released from exosomes to the outside of the cell to support tumor cell proliferation and tumor growth. Kanlikilicer et al. (2016) reported that intracellular *miRNA-6126* expression was downregulated in ovarian carcinoma cells *via* exosomal transport, thus stimulating the expression of the target gene *ITGB1* (integrin subunit *β*1) as fuel to cancer cell proliferation and invasion. Their findings support our presumption. However, the precise mechanisms for exosomal secretion of nucleic acid into the circulation remain uncertain. Future studies are still needed to explore how the nuclear transcription factor *BATF2* migrates from the nucleus to the cytoplasm and how a *BATF2*-positive exocytic exosome forms.

This study for the first time reported serum and exosomal *BATF2* mRNA expressions in NPC patients, with positive rates of 51.47 and 48.52%, respectively. Their high AUCs in discriminating NPC from healthy controls and high sensitivity in identifying recurrent patients from treated ones suggest a high potential for its clinical application in predicting diagnosis and monitoring efficacy and post-treatment relapse for patients. They are expected to serve as new biomarkers in NPC clinical practice. Our previous study series reported similar performances of *LDHC*/LDH-C4, a cancer-testis antigen, in diagnosis and relapse prediction and efficacy evaluation for breast cancer and hepatocellular carcinoma patients (Cui et al., 2020; Cui et al., 2020). As the total exosomal RNA is part of the total serum RNA extracted using an exoRNeasy Serum/Plasma Midi Kit, the expression score of serum *BATF2* mRNA must be higher than that of exosomal *BATF2* mRNA. This finding indicates a significant resource of cell-free *BATF2* molecules in serum or plasma from exosomal *BATF2*, which is consistent with our previous results (Cui et al., 2020; Cui et al., 2020). However, this conclusion needs further validation. Current limitations to clinical application of exosomal gene detection mainly ascribe to complicated procedures to enrich and purify exosomal DNAs/RNAs. Our conclusion is also limited by sample size and a lack of multiple data collection strategies, which can be solved by implementing multicenter clinical trials with a large sample size in the future.

## Conclusion

This study explored the expressions of BATF2 mRNA and protein in NPC patients’ tissues, serum, and serum-derived exosomes. BATF2/*BATF2* is expressed cancerous tissues, serum, and serum-derived exosomes in NPC patients. Circulating and tissue *BATF2* mRNA can serve as a multipurpose biomarker capable of the diagnosis, prognosis prediction, efficacy evaluation, and recurrence monitoring in NPC.

## Data Availability

The raw data supporting the conclusion of this article will be made available by the authors, without undue reservation.
